# Impact of sleep-wake features on fatigue among female shift work nurses

**DOI:** 10.1080/07853890.2023.2210843

**Published:** 2023-05-17

**Authors:** Xin Zhang, Xuesong Dai, Jing Jiao, Shih-Yu Lee

**Affiliations:** a School of Nursing, Chinese Academy of Medical Sciences and Peking Union Medical College, Beijing, China; b Department of Nursing Quality Control, Beijing Hospital, Beijing, China; c Department of Nursing, Peking Union Medical College Hospital, Beijing, China; d School of Nursing, Hungkuang University, Taichung, Taiwan, China

**Keywords:** Circadian activity rhythms, reaction time, cortisol, fatigue, shift work

## Abstract

**Background:**

Sleep disturbance and fatigue are prevalent in nurses. Little is known about the characteristics of shift work nurses’ sleep-wake features and their subsequent impact on work performance. The study aimed to describe the characteristics of the sleep-wake index, reaction time, saliva cortisol level, and fatigue severity among female shift work nurses.

**Methods:**

This is a cross‐sectional exploratory study. A convenience sample of 152 female nurses (8-hour day-evening-night, *n* = 70; 12-hour day–night, *n* = 82) participated in this study from nine intensive care units (ICUs) from two teaching hospitals in Beijing, China. A consecutive 7-day actigraphy data were used to analyse sleep-wake indexes, including total sleep time (TST) and circadian activity rhythms (CAR). Before and after shifts, the following data were collected, psychomotor vigilance task for reaction time, saliva cortisol for the level of alertness, and self-reported fatigue severity with the Lee Fatigue Scale-Short Form.

**Results:**

All nurses reported clinically significant fatigue severity. Compared with the 8-hour shift nurses, the 12-hour shift nurses had significantly more TST (456 vs. 364 min), higher saliva cortisol levels before the day shift (0.54 vs. 0.31), but longer reaction time before the night shift (286 vs. 277 ms). In both shifts, those with better CAR had significantly longer TST.

**Conclusion:**

Female nurses experienced fatigue and desynchronized CAR, especially nurses on a 12-hour shift. The CAR-friendly shift work schedule is needed to minimize the health and safety impacts of circadian misalignment for nurses.

**Key messages**
- This is the first use of consecutive 7-day actigraphy data to explore the link between sleep disturbances as a stressor to CAR, salivary cortisol, and reaction time among clinical nurses.
- CAR may be a helpful indicator for overworked nurses, and it can serve as a modifiable target for interventions to enhance nurses’ well-being.

## 1. Introduction

Shift work is working during periods other than the day shift from 8 am to 5 pm [1]. It can cause circadian rhythm disruption as work interferes with an individual’s standard night-time sleep schedule [2]. Circadian misalignment is related to sleep restriction, irregular endogenous circadian rhythms, sleep-wake cycles, and external environmental cues [2]. Evidence shows that the impact of circadian misalignment exceeds the effect of sleep disruption; therefore, shift workers may not quickly recover from irregular sleep-wake patterns and may adversely affect safety and health [3,4]. Shift work nurses must frequently modify their daily routines due to the changes in their work and sleep schedules associated with each shift rotation.

Studies found that shift workers who adapted well to their schedule had less sympathetic nerve activity during sleep, which resulted in longer sleep time, better performance, alertness, and mood [5,6]. However, being well-adapted to shift work is rare. A meta-analysis concluded that only 3% of permanent night workers fully adapted to their schedule, and there were individual differences in the ability to adjust to shift work [7]. For example, gender plays a role; female nurses exhibited high stress levels and experienced more sleep and mental problems than their male counterparts [8,9]. Therefore, it is essential to explore the effect of shift work on women’s health, work performance, and cognitive function since they comprise most of the nursing workforce. In addition, in China, the rapidly rotating shift is mandatory for nurses. They alternate day, evening, and night shifts within one week [10], which might create more challenges for nurses in China.

Nearly one decade ago, researchers pointed out that the rapidly rotating shift is challenging to adapt to while minimizing circadian misalignment; therefore, they recommended eliminating this kind of work schedule [11]. However, the rapidly rotating shift schedules are still commonly used in clinical nursing practice in China, Korea, and Italy [6,10,12]. Such a work schedule can increase fatigue severity, poor sleep, decreased work performance [6], and impaired memory [13]. A systematic review concluded that rapidly rotating shift work could lead to fragmented sleep, decreased total sleep time, and increased fatigue severity [14]. More than one decade ago, researchers suggested that the optimal working schedule should limit night shifts to three nights and allow three days off before resuming changes [15]. Today, hospitals in many countries have implemented a 12-hour shift system to optimize work arrangements. It is well-known that day shifts are safer than night shifts because night shifts require extended rest periods to regain energy [16]. However, physiological evidence regarding the effects of different shift work hours is lacking.

Circadian rhythms synchronize human functions to the 24 h a day cycle in the rest-activity pattern. Studies have shown that desynchronized circadian rhythms can negatively impact health, affecting key central nervous system regulation pathways [17]. Circadian activity rhythms (CAR) is the ratio between peak activity (amplitude) and adjusted mean activity [18]. CAR is a surrogate metric of internal circadian rhythms assessed by actigraphy, and CAR is a useful biological marker for internal circadian rhythms in humans [19]. To date, little is known about the characteristics of nurses’ CAR, especially those who work in rapidly rotating shifts, and their subsequent impact on work performance.

The effectiveness of work performance may be related to the individual’s alertness and attention level. The psychomotor vigilance task (PVT) is commonly used to measure the lack of alertness and sustained attention [20]; lack of sleep is associated with slower reaction times and increased lethargy [21]. From the physiological perspective, the cortisol level indicates alertness since it is an effective stress indicator, which is affected by circadian rhythms and coping mechanisms [8].

Sleep disturbances could trigger a stress response with a subsequent activation of the psychoneuroimmunological (PNI) pathway that impacts health and work performance [22]. Rober Ader introduced the PNI concept in 1980 [22]. PNI focuses on the associations between mental processes and health by examining the interaction between psychological function and the body’s nervous, endocrine, and immune systems. Psychological stress triggers the autonomic nervous system and the endocrine responses resulting in a fight-or-flight reaction. Repetition of this process due to chronicle stress over a long period leads to wear-and-tear adverse impacts on physical and mental conditions [22,23]. The most immediate observable consequence of such adverse effects is sleep disturbances resulting from inadequate total sleep time or sleep disruption. Studies exploring the link between sleep disturbances as a stressor to CAR, salivary cortisol, and work performance are rare. Based on the PNI theory [22], behavioural (i.e. shift work), neurological (i.e. alertness), and endocrine (i.e. cortisol) processes of adaptation have an impact on health.

## 2. Aim

The current study aimed to describe the characteristics of sleep-wake indexes (total sleep time, wake after sleep onset [WASO], CAR), reaction times, saliva cortisol level, and fatigue severity among 8- and 12-hour rapidly rotating shift work female nurses.

## 3. Methods

### 3.1. Study design and participants

This cross-sectional exploratory study used a convenience sample of nine intensive care units (ICUs) from two teaching hospitals in Beijing, China: four ICUs and all nurses on 8-hour shifts in one hospital, and another five ICUs in another, all nurses work 12-hour shifts. The sample size was calculated using G-power. A minimum sample size of 146 (73 in each shift length group) would be needed to achieve 85% power in a two-tailed test to detect the medium effect size. The inclusion criteria were ICU female nurses working rapid shifts (rotating day, evening, and night shifts within one week). The two hospitals routinely use a shift system. The 8-hour shift is day-evening-night-off-day-day-off, and the 12-hour shift is day-night-off-off-night-off-off. For shift schedule, the 12-hour shift is 07:45–19:45 and 19:45–7:45; the 8-hour shift is day shift 08:00–16:00/08:00–12:00 and 14:00–18:00; the evening shift 18:00–02:00, and the night shift 02:00–08:00. The nurses who self-reported pregnant, with a history of obstructive sleep apnea (which may give a higher waking time from the overnight actigraphy monitoring), and mental illness (e.g. anxiety disorder, depressive symptom) were excluded. The study was approved by the Institutional Review Board at the research site (No. 201612028).

The nursing department sent recruitment emails through WeChat (a common social media in China). Due to the limited supply of actigraphy, we could only enroll 20 nurses every two weeks for the objective sleep data collection. Potential participants were informed that their participation was entirely voluntary. From January to November 2020, a total of 173 female nurses were enrolled in this study, and all signed informed consent; however, 21 participants were excluded for either having their actigraphy recording less than seven days (*n* = 6), or their saliva samples (*n* = 15) were not suitable for analysis. Therefore, a total of 152 participants (response rate= 87.86%, 82 in the 12-hour shift and 70 in the 8-hour shift) were included in the final analysis.

### 3.2. Measures

A demographics form was used to gather the participant’s age, height and weight (for BMI), education, marital status, and shift work rotation. The link between BMI and sleep has been controversial [24]; we used it as a confounding variable in this study. Objective data (sleep-wake index, reaction times, and salivary cortisol) and subject fatigue data were collected as described below.

Actigraphy (AMI: Ambulatory Monitoring, Inc., Ardsley, New York), a non-invasive motion sensor monitor, assessed the sleep-wake indexes (total sleep time, CAR). Various researchers have used polysomnographic measures of sleep to validate wrist actigraphy as a measure of total sleep time and wake during the night (*r*= .93 to .99) [18,25]. Nurses wore the actigraphy on their non-dominate hand for a consecutive seven days (a period of their rapidly rotating shift). Study participants were asked to keep a 7-day sleep diary to record their bedtime and arise time, which was used to validate the event markers from the actigraphy data. The mean values of TST and WASO for seven nights and the standard error of the mean were used for data analysis. The rest-activity patterns, mesor (24-hour adjusted activity mean) and amplitude (mesor to peak) were used to calculate the circadian quotient (amplitude/mesor) to indicate CAR [18]. A CAR value close to one suggests a more robust and synchronized rest-activity pattern.

The PVT-192 device (Ambulatory Monitoring, Inc.), a handheld and self-contained system, assessed reaction times. The PVT-192 was delivered a three-mm visual stimulus (a reaction-time counter), which requires the participant to make a pushbutton response within 1.5 s with an inter-stimulus interval varying from 2 to 10 s [26]. Participants were instructed to respond to a visual stimulus presented at various intervals (random, 2,000–10,000 ms), over 5 min, by pressing the button on the device; however, no cut-off point was suggested. Four sets of PVT data were collected from each participant before and after day- and night-shifts at a private and quiet lounge in the ICU. The nurse was instructed to avoid caffeine, alcohol, and over-the-counter medications before the test; each participant had a 1-minute training.

Since salivary cortisol is highly correlated with blood cortisol levels, it is often used in studies on circadian rhythm and work-related stress [27,28]. To enhance the validity of the salivary sampling, the researchers used the manufacturer’s standardized protocol for sample collection, transportation, storage, and analysis [29]. Morning saliva samples were collected between 7:00 and 8:00, and night samples were collected between 19:00 and 20:00 at a private and quiet lounge in the ICU. The nurses rinsed their mouths with tap water, then 30 min later, deposited 2 ml of saliva in a sterile labeled plastic tube. Samples were stored at −20 °C then assessed using a Liquid chromatography-mass spectrometer (LC-MS/MS, 4000QTRAP, AB SCIEX, USA). Morning salivary cortisol levels were categorized as the normal range (0.094 − 1.551 μg/dL), below the normal range (< 0.094 μg/dL), and above the normal range (> 1.551 μg/dL); night-time levels were categorized as the normal range (≤ 0.359 μg/dL) and above the normal range (> 0.359 μg/dl) [8,30].

Fatigue severity was measured with the Lee Fatigue Scale-Short Form (C-LFS-SF) [31]. The level of fatigue should be affected by the level of alertness of the individual; therefore, the severity of fatigue was assessed before the four PVT measurements. C-LFS-SF is a self-rated, 8-item scale, rating from 0 (not fatigued) to 10 (extremely fatigued), and is adapted from the Lee Fatigue scale [32], which includes a 13-item fatigue subscale and a 5-item energy subscale. A mean score of 3.3 or higher indicates clinically significant fatigue. The Cronbach’s alpha for the C-LFS-SF is 0.97 and has good construct validity [31].

### 3.3. Data analysis

The automatic sleep-scoring program (Action 4 Software Program, AMI) calculated TST in minutes and WASO as a percentage of the night’s time in bed trying to sleep. Data were analysed by using SPSS 24.0 version. Before analysis, all data were evaluated to ensure that all met statistical assumptions. Descriptive statistics were used to describe sample characteristics and the major variables, including mean ± SD, median (P_25_, P_75_), range, and percent. T-tests were used for the post-hoc comparisons between the means, Mann-Whitney tests were used for the non-parametric equivalent of the independent samples t-test, and chi-square tests were used to test group differences in the categorized data.

## 4. Results

### 4.1. Participant characteristics

The 152 participants’ mean age was 31.8 (SD = 3.1) years (Table 1). They were either working 8- (46.1%) or 12-hour shifts, and all rotated day and night shifts within one week. The majority of them had a normal BMI (69.7%); however, 13 (8.6%) were underweight, and 33 (21.7%) were either overweight or obese. Compared with the 12-hour shift nurses, the 8-hour shift nurses had more ICU experience and higher patient loads.

**Table 1.** Nurses’ demographic and working characteristics (*N* = 152).

Variable	Mean **(**SD)/*N* (%)	12h (*n* = 82)	8h (*n* = 70)	Comparison	
		Mean **(**SD)/*N* (%)	Mean **(**SD)/*N* (%)	*X^2^*/ t/Z	*p* Value
Age (years)	31.81 ± 6.09	31.81 ± 5.28	31.81 ± 6.97	−0.009	0.992
BMI	21.99 ± 3.29	22.05 ± 3.53	21.91 ± 3.01	0.249	0.804
Education level				6.928	0.074
Associate’s	30(19.7)	10(12.2)	20(28.6)		
Bachelor’s and above	122(80.3)	70(87.8)	50(71.4)		
Marital status				0.373	0.541
Married	93(61.2)	30(36.6)	41(58.6)		
Single	59(38.8)	52(63.4)	29(41.4)		
Years worked in the hospital^a^	10(4, 16)	10(5, 15.25)	9.5(3, 16.25)	−0.248	0.804
Years worked in the ICU^a^	7(2, 11.75)	4.5(2, 10)	8(3.00, 15.25)	−2.346	0.019
Nurse-patient ratio	3.85 ± 1.25	3.24 ± 0.95	4.56 ± 1.18	−7.441	0.000

^a^All are the median (P_25_, P_75_).

### 4.2. Characteristics of the sleep-wake index, reaction times, saliva cortisol level, and fatigue severity among 8- and 12-hour shifts nurses

Table 2 detailed the characteristics and differences between two rapidly rotating shift work nurses. Compared with 8-hour shift nurses, the average total sleep time of 12-hour shift nurses was significantly higher (12-hour= 455.64 vs. 8-hour= 363.98 min). A synchronized CAR should be close to one; however, for nurses in 8- or 12-hour shifts, their CAR is desynchronized (12-hour = 0.53 vs. 8-hour = 0.50). Compared with the 8-hour shift nurses, the saliva cortisol level of 12-hour shift nurses was significantly higher before the day shift (median 0.54 vs. 0.31, *p* < .005) and after the night shift (median 0.51 vs. 0.31, *p* < .005), and the reaction time was longer before the night shift (288.46 vs. 268.99 ms, *p* = .033). As for fatigue, all the nurses experienced clinically significant fatigue severity regardless of whether before or after their shift work; however, compared to the 8-hour group, the 12-hour group reported significantly lower fatigue severity before the day and night shifts (*p* < .001).

**Table 2.** Characteristics and differences in the sleep-wake index, reaction times, saliva cortisol level, and fatigue severity among 8- and 12-hour shifts nurses (*N* = 152).

	12h (*n* = 82)	8h (*n* = 70)	Comparison	
Variable	Mean **(**SD)/*N* (%)	Mean **(**SD)/*N* (%)	*X^2^*/ t/Z	*p* Value
**Actigraphy**				
TST (min)	455.64 ± 97.30	363.98 ± 119.97	5.200	0.000
WASO (%)^a^	3.86 (1.60, 7.23)	2.78 (1.30, 5.72)	−1.148	0.251
CAR	0.53 ± 0.13	0.50 ± 0.18	1.278	0.203
Salivary cortisol^a^				
Before day shift	0.535 (0.246, 1.045)	0.312 (0.152, 0.633)	−3.094	0.002
Abnormal	14 (17.1)	17 (24.3)		
After day shift	0.202 (0.122, 0.456)	0.182 (0.072, 0.335)	−1.643	0.100
Abnormal	25 (30.5)	15 (21.4)		
Before night shift	0.216 (0.123, 0.494)	0.194 (0.053, 0.463)	−1.697	0.090
Abnormal	29 (35.4)	19 (27.1)		
After night shift	0.513 (0.262, 0.894)	0.313 (0.134, 0.619)	−2.778	0.005
Abnormal	10 (12.2)	16 (22.9)		
Reaction time mean				
Before day shift	321.55 ± 71.32	314.26 ± 72.54	0.623	0.534
After day shift	335.77 ± 88.09	310.30 ± 72.46	1.754	0.082
Before night shift	317.00 ± 76.04	303.34 ± 62.69	1.195	0.234
fter night shift	345.47 ± 93.63	357.03 ± 101.63	−0.729	0.467
Reaction time median				
Before day shift	285.61 ± 57.37	277.04 ± 51.45	0.962	0.337
After day shift	303.25 ± 76.87	281.99 ± 60.42	1.872	0.063
Before night shift	288.46 ± 67.81	268.99 ± 36.42	2.150	0.033
After night shift	313.16 ± 74.23	308.51 ± 69.60	0.394	0.694
Fatigue severity				
Before day shift	3.78 ± 2.26	4.76 ± 1.96	−2.816	0.006
After day shift	6.99 ± 2.13	5.99 ± 1.84	3.064	0.003
Before night shift	4.04 ± 2.55	6.42 ± 2.06	−6.267	0.000
After night shift	8.14 ± 1.88	7.62 ± 1.71	1.784	0.076

^a^All are the median (P_25_, P_75_).

TST: total sleep time; WASO: wake time after sleep onset; CAR: circadian activity rhythm.

### 4.3. Differences in the two groups according to the median of CAR

To date, no existing data is available to determine the cut-off point of CAR values. Therefore, the median split (.53 for 12-hour shift and .50 for 8-hour shift) was used to divide the nurses into two groups, poor CAR and good CAR, for further analysis. For nurses on the 12-hour shift, compared with nurses with poor CAR, those with better CAR were younger, single, worked fewer years in the hospital, and had fewer night shifts in one week (Table 3). For both 8- and 12-hour shifts, nurses with better CAR had longer total sleep time. Although all nurses reported clinically significant fatigue severity, there was no significant difference between the good and poor CAR groups.

**Table 3.** Differences in the two groups according to the median of CAR (*N* = 152).

Variable	**Mean (SD)/***N* (%)	**Mean (SD)/***N* (%)	*X^2^*/ t/Z	*p* Value
12-hour (*n* = 82)	CAR ≤ 0.53 (*n* = 38)	CAR > 0.53 (*n* = 44)		
Age (years)	33.55 ± 5.19	30.30 ± 4.92	2.914	0.005
Marital status			5.080	0.024
Married	29 (76.3)	23 (52.3)		
Single	9 (23.7)	21 (47.7)		
Years worked in the hospital^a^	10 (5, 15.25)	9.5 (3, 16.25)	−2.436	0.015
TST (min)	427.76 ± 95.20	479.73 ± 93.57	−2.488	0.015
WASO (%)^a^	6.47 (1.99,10.89)	2.85 (1.11, 5.48)	−3.050	0.002
Subjective fatigue				
Before day shift	4.27 ± 2.27	3.36 ± 2.19	1.849	0.068
After day shift	7.41 ± 2.07	6.62 ± 2.11	1.712	0.091
Before night shift	3.98 ± 2.69	4.09 ± 2.45	−0.199	0.843
After night shift	8.30 ± 2.00	3.21 ± 0.93	0.713	0.478
8-hour (*n* = 70)	CAR ≤ 0.50 (*n* = 33)	CAR > 0.50 (*n* = 37)		
Nurse-patient ratio	4.88 ± 1.11	4.27 ± 1.19	2.199	0.031
TST (min)	330.73 ± 134.48	393.65 ± 97.95	−2.254	0.027
Subjective fatigue				
Before day shift	4.72 ± 2.06	4.79 ± 1.90	−0.153	0.879
After day shift	5.94 ± 2.17	6.03 ± 1.53	−0.204	0.839
Before night shift	6.01 ± 2.13	6.79 ± 1.94	−1.591	0.116
After night shift	7.41 ± 1.74	7.80 ± 1.69	−0.966	0.338

^a^All are the median (P_25_, P_75_).

TST: total sleep time; WASO: wake time after sleep onset; CAR: circadian activity rhythm.

## 5. Discussion

Based on the PNI theory [22], this study used consecutive 7-day actigraphy data to explore the link between sleep disturbances as a stressor to CAR, salivary cortisol, and work performance among clinical nurses. To our best knowledge, this is the first report in this area. Nurses in this study had problems with both sleep and CAR. On average, they slept fewer than recommended 8 h, which is especially problematic for nurses on 8-hour shifts. In the current study, the total sleep time of 8-hour shift nurses was significantly less than that of 12-hour shift nurses, and they also reported higher levels of fatigue at the beginning of day and night shifts. WASO of more than 10% indicates sleep is fragmented and of poor quality [18]. Although the WASO of nurses in this study was within the normal range, this may be the result of extreme fatigue and difficulty in waking up at night, especially for the 8-hour shift nurses. This might be related to the 8-hour shift nurses working on three different shifts (day-evening-night) in the roll, only taking one day then returning for two more days; however, for the 12-hour shift, they work two shifts (day and night), take two days off, then work another night shift then take two days off. Compared with the 8-hour shift nurses, the 12-hour nurses have more time off to recover. Burgess [15] suggested that a minimum of 3 days off is required between shifts changing. Prior studies found a high correlation between short total sleep time and nurse fatigue [33,34]; therefore, nursing managers should be familiar with the sleep mechanism and consider it nurse when arranging schedules for nurses.

None of the participants in this study were allowed to have enough time to recover their circadian rhythms. Their rest-activity rhythms were desynchronized and evidenced by low CAR (<.53), which was similar to the index of older people with cognitive impairment [35]. Therefore, the long-term effects of rapidly rotating shift work on psychological and cognitive function should be monitored, especially for those middle-age nurses with sleep problems. In addition, the start time of night shifts may be another issue worthy of further discussion. Melatonin production generally starts around 7-8 pm and peaks around 2-3 am [36]. In the current study, the shift changed at 19:45 for the 12-hour shift and at 2 am for the 8-hour shift, which may negatively impact nurses’ circadian rhythm adaptation and work performance. Therefore, examining the nurse’s desynchronized CAR associations with cognitive function was necessary. CAR may be a helpful indicator of when nurses are overworked and serve as a modifiable target for interventions to enhance the nurse’s well-being.

The average fatigue scores of the current study participants in all shifts were above the cut-off point, indicating that they experienced clinically significant fatigue severity and needed attention. Shift work nurses are vulnerable to workplace fatigue, which is a physiologically impaired work state caused by four main factors: sleep loss (acute and chronic), prolonged awake time (more than 16 h), working at suboptimal times in the circadian master clock cycle, and physical and mental work overload [37]. Findings from the current study show that rapid-shift work nurses have desynchronized CAR and workplace fatigue, which is indicated by the self-report and prolonged reaction time; these two issues may negatively affect each other and patient safety. Further, chronic fatigue may lead to the accumulation of DNA damage and increase the incidence of cancer and other diseases [38]; therefore, this issue needs to be addressed.

Workplace stress has been recognized globally as a risk factor affecting the health and safety of workers [8,39]. In the current study, 20.4% of the study participants had abnormal cortisol levels before the day shift and 17.1% after the night shift, indicating they were either too alert to fall asleep after the night shift or not alert enough during the day shift to avoid errors. Longer shift duration (more than 8 h) was significantly associated with impaired morning cortisol levels [8]; therefore, the findings support the need to examine shift patterns and stress-coping strategies to promote nurses’ health and maintain workplace safety. Alertness is essential during work to minimize the risk of accidents and promote healthcare workers’ safety. Compared with nurses who only work day- or night-shifts [5], our study participants have longer reaction times, which suggests that rapid shift work may harm cognition and response and deserves further attention. Therefore, examining workplace stress’s associations with alertness was necessary. The reaction time may indicate the nurse’s ability to provide competent clinical practice. Nursing administrators should design the organizational interventions (e.g. CAR-friendly rotation schedules, individual shift work tolerance, objective measures of vigilance and sleepiness, and a low-risk environment) and personalized guidance for shift nurses to increase their capacity for doing time and task management, especially nurses on 12-hour shift [40].

Despite these promising findings, the study has several limitations. Findings should be considered in light of the only-once collection time point of reaction times, cortisol, and subjective fatigue in the first of day and night shifts, which may threaten internal validity. The nurses were only drawn from ICUs from two teaching hospitals, and only bivariate analyses were conducted for the hospital characteristics. Although we used PNI to guide this study, the immunology biomarkers were not included in the current study due to the technology limitation in our lab. We did not directly evaluate the presence of sleep quality through specific questionnaires. Other endocrines markers, such as melatonin and estradiol, should also be collected to explore further the role of biomarkers related to health outcomes.

## 6. Conclusion and implications for practice

Female nurses experienced fatigue and poor CAR in rapid-shift rotation. The results support the potential of circadian-based interventions to minimize circadian misalignment’s health and safety impacts. Circadian rhythm-based interventions should discuss shift length, speed of shift rotation, and number and duration of consecutive shifts, in addition, to emphasizing the importance of CAR and its association with cognitive function. Further research should be based on a longitudinal design with larger sample size and repeated measurement to understand asynchronized CAR over time and determine causal relationships between variables in nurses with shift work. Another suggestion is to conduct a comparative study for the sub-group analysis between participants with high-CAR and low-CAR value affiliations to establish and develop the role of CAR as a coping strategy to deal with health problems of different age groups. To ensure the internal validity of the study findings, self-reported data, including qualitative interviews, should be considered in addition to objective data in future studies.

## Data Availability

Data is available on request from the authors.

## References

[1] Canadian Centre for Occupational Health & Safety. Rotational shiftwork. 2017. www.ccohs.ca/oshanswers/ergonomics/shiftwrk.html

[2] Scheer FA, Hilton MF, Mantzoros CS, et al. Proceedings of the national academy of sciences of the USA. Curr Boil: CB. 2009;106:1–8. 10.1073/pnas.0808180106 PMC2657421 19255424

[3] Morris CJ, Purvis TE, Mistretta J, et al. Circadian misalignment increases C-reactive protein and blood pressure in chronic shift workers. J Biol Rhythms. 2017;32(2):154–164. 28347188 10.1177/0748730417697537 PMC5858578

[4] Chellappa SL, Vujovic N, Williams JS, et al. Impact of circadian disruption on cardiovascular function and disease. Trends Endocrinol Metab. 2019;30(10):767–779. 31427142 10.1016/j.tem.2019.07.008 PMC6779516

[5] Behrens T, Burek K, Pallapies D, et al. Decreased psychomotor vigilance of female shift workers after working night shifts. PLoS One. 2019;14(7):e0219087. 31276523 10.1371/journal.pone.0219087 PMC6611661

[6] Di Muzio M, Reda F, Diella G, et al. Not only a problem of fatigue and sleepiness: changes in psychomotor performance in italian nurses across 8-h rapidly rotating shifts. JCM. 2019;8(1):47. 30621274 10.3390/jcm8010047 PMC6352064

[7] Folkard S. Do permanent night workers show circadian adjustment? A review based on the endogenous melatonin rhythm. Chronobiol Int. 2008;25(2):215–224. 18533325 10.1080/07420520802106835

[8] Bani-Issa W, Radwan H, Al Marzooq F, et al. Salivary cortisol, subjective stress and quality of sleep among female healthcare professionals. J Multidiscip Healthc. 2020;13:125–140. 32103972 10.2147/JMDH.S229396 PMC7008192

[9] Ding M, Lawson C, Johnson C, et al. Occupational exposure to high-level disinfectants and risk of miscarriage among nurses. Occup Environ Med. 2021;78(10):731–737. 34039757 10.1136/oemed-2020-107297 PMC8634624

[10] Zhang X, Lee SY, Luo H, et al. A prediction model of sleep disturbances among female nurses by using the BP-ANN. J Nurs Manag. 2019;27(6):1123–1130. 31006161 10.1111/jonm.12782

[11] Smith MR, Eastman CI. Shift work: health, performance and safety problems, traditional countermeasures, and innovative management strategies to reduce circadian misalignment. Nat Sci Sleep. 2012;4:111–132. 23620685 10.2147/NSS.S10372 PMC3630978

[12] Garde AH, Harris A, Vedaa Ø, et al. Working hour characteristics and schedules among nurses in three nordic countries - a comparative study using payroll data. BMC Nurs. 2019;18:12. 30962763 10.1186/s12912-019-0332-4 PMC6438001

[13] Thun E, Waage S, Bjorvatn B, et al. Short sleep duration and high exposure to quick returns are associated with impaired everyday memory in shift workers. Nurs Outlook. 2021;69(3):293–301. 33127075 10.1016/j.outlook.2020.09.008

[14] Vedaa Ø, Harris A, Bjorvatn B, et al. Systematic review of the relationship between quick returns in rotating shift work and health-related outcomes. Ergonomics. 2016;59(1):1–14. 26072668 10.1080/00140139.2015.1052020

[15] Burgess PA. Optimal shift duration and sequence: recommended approach for short-term emergency response activations for public health and emergency management. Am J Public Health. 2007;97(Supplement_1):S88–S92. 17413074 10.2105/AJPH.2005.078782 PMC1854972

[16] Gander P, O’Keeffe K, Santos-Fernandez E, et al. Development and evaluation of a matrix for assessing fatigue-related risk, derived from a national survey of nurses’ work patterns. Int J Nurs Stud. 2020;112:103573. 32334846 10.1016/j.ijnurstu.2020.103573

[17] Serin Y, Acar Tek N. Effect of circadian rhythm on metabolic processes and the regulation of energy balance. Ann Nutr Metab. 2019;74(4):322–330. 31013492 10.1159/000500071

[18] Ancoli-Israel S, Cole R, Alessi C, et al. The role of actigraphy in the study of sleep and circadian rhythms. Sleep. 2003;26(3):342–392. 12749557 10.1093/sleep/26.3.342

[19] Ortiz-Tudela E, Iurisci I, Beau J, et al. The circadian rest-activity rhythm, a potential safety pharmacology endpoint of cancer chemotherapy. Int J Cancer. 2014;134(11):2717–2725. 24510611 10.1002/ijc.28587

[20] Dinges DF, Pack F, Williams K, et al. Cumulative sleepiness, mood disturbance, and psychomotor vigilance performance decrements during a week of sleep restricted to 4-5 hours per night. Sleep. 1997;20:267–277. 9231952

[21] Wilson M, Permito R, English A, et al. Performance and sleepiness in nurses working 12-h day shifts or night shifts in a community hospital. Accid Anal Prev. 2019;126:43–46. 28987265 10.1016/j.aap.2017.09.023

[22] Ader R. On the development of psychoneuroimmunology. Eur J Pharmacol. 2000;405(1-3):167–176. 11033324 10.1016/s0014-2999(00)00550-1

[23] Chang WP, Wang CH. Influence of sleep fragmentation and fatigue on turnover of female nurses working rotating shifts. J Clin Nurs. 2022;31(23-24):3573–3583. 34957611 10.1111/jocn.16184

[24] Garfield V. The association between body mass index (BMI) and sleep duration: where are we after nearly two decades of epidemiological research? IJERPH. 2019;16(22):4327. 31698817 10.3390/ijerph16224327 PMC6888565

[25] Jean-Louis G, von Gizycki H, Zizi F, et al. Determination of sleep and wakefulness with the actigraph data analysis software (ADAS). Sleep. 1996;19:739–743. 9122562

[26] Dinges DF, Powell JW. Microcomputer analyses of performance on aportable, simple visual RT task during sustained operations. Behav Res Methods Instrum Comput. 1985;17(6):652–655.

[27] Baba M, Ohkura M, Koga K, et al. Analysis of salivary cortisol levels to determine the association between depression level and differences in circadian rhythms of shift-working nurses. J Occup Health. 2015;57(3):237–244. 25752657 10.1539/joh.14-0079-OA

[28] An K, Salyer J, Brown RE, et al. Salivary biomarkers of chronic psychosocial stress and CVD risks: a systematic review. Biol Res Nurs. 2016;18(3):241–263. 26349905 10.1177/1099800415604437

[29] Salimetrics LLC, SalivaBio LLC. Saliva collection and handling advice. Available from:http://www.salimetrics.com. Accessed December 1, 2019.

[30] Aardal E, Holm AC. Cortisol in saliva-reference ranges and relation to cortisol in serum. Eur J Clin Chem Clin Biochem. 1995;33(12):927–932. 8845424 10.1515/cclm.1995.33.12.927

[31] Tsai SY, Shun SC, Lai YH, et al. Psychometric evaluation of a chinese-version of the lee fatigue scale-short form in women during pregnancy and postpartum. Int J Nurs Stud. 2014;51(7):1027–1035. 24262779 10.1016/j.ijnurstu.2013.10.023

[32] Lee KA, Hicks G, Nino-Murcia G. Validity and reliability of a scale to assess fatigue. Psychiatry Res. 1991;36(3):291–298. 2062970 10.1016/0165-1781(91)90027-m

[33] Kecklund G, Axelsson J. Health consequences of shift work and insufficient sleep. BMJ. 2016;355:i5210. 27803010 10.1136/bmj.i5210

[34] Bruyneel A, Smith P, Tack J, et al. Prevalence of burnout risk and factors associated with burnout risk among ICU nurses during the COVID-19 outbreak in french speaking Belgium. Intensive Crit Care Nurs. 2021;65:103059. 33875341 10.1016/j.iccn.2021.103059 PMC9759739

[35] Ancoli-Israel S, Klauber MR, Jones DW, et al. Variations in circadian rhythms of activity, sleep, and light exposure related to dementia in nursing-home patients. Sleep. 1997;20:18–23. 9130329

[36] Lee KA. Impaired sleep, in pathophysiological phenomena in nursing. Saunders: St. Louis 2003. 363–385.

[37] International Civil Aviation Organization. Manual for the oversight of fatigue management approaches, 2nd ed. 2016. Available from: https://www.icao.int/safety/fatiguemanagement/pages/resources.aspx.

[38] Potter GDM, Wood TR. The future of shift work: circadian biology meets personalised medicine and behavioural science. Front Nutr. 2020;7:116. 32850937 10.3389/fnut.2020.00116 PMC7426458

[39] Mattila E, Peltokoski J, Neva MH, et al. COVID-19: anxiety among hospital staff and associated factors. Ann Med. 2021;53(1):237–246. 33350869 10.1080/07853890.2020.1862905 PMC7877952

[40] Alfonsi V, Scarpelli S, Gorgoni M, et al. Sleep-related problems in night shift nurses: towards an individualized interventional practice. Front Hum Neurosci. 2021;15:644570. 33796014 10.3389/fnhum.2021.644570 PMC8007770

